# Isolated bronchial hemangioma causing recurrent hemoptysis: A case report

**DOI:** 10.1097/MD.0000000000036135

**Published:** 2023-11-17

**Authors:** Zhipeng Lin, Xugong Zou, Xiaolong Hu, Yuan Chen, Xiaoqun Li, Dabei Huang, Jian Zhang

**Affiliations:** a Department of Interventional Medicine, Zhongshan People’s Hospital, Guangdong, China.

**Keywords:** bronchial hemangioma, interventional therapy, recurrent hemoptysis

## Abstract

**Rational:**

The development of bronchial hemangioma in adults is rare, and massive hemoptysis due to diffuse vascular proliferation of bronchial hemangioma is fatal.

**Patient concerns:**

A case of a 29-year-old woman kept massive hemoptysis even after being underwent repeated interventional embolization for recurrent massive hemoptysis. Eventually, the patient was performed the operation of right upper lung lobectomy and bronchial hemangioma with extracorporeal membrane oxygenation support and was followed up for 4 years without recurrent hemoptysis.

**Diagnoses:**

Bronchial hemangioma.

**Conclusion:**

For patients with bronchial angiomas bonded with bronchial artery-pulmonary arteriovenous fistulae, the early surgical resection is recommended if bronchial artery embolization (BAE) is considered ineffective.

## 1. Introduction

Bronchial hemangioma is a very rare benign tumor, mostly due to congenital abnormal vascular development. There are few reported cases of bronchial hemangioma worldwide, and there is still a lack of research on its pathogenesis. Most scholars now believe that it is closely related to angiogenesis.^[1]^ Sweetser classified tracheal hemangiomas into infantile and adult types.^[2]^ Angiomas of the trachea and bronchi in adults are extremely rare.

The diagnosis of bronchial hemangioma is mainly based on CT or CTA, fiberoptic bronchoscopy, and histopathological examination. At present, most of the reports make the diagnosis based on pathology suggestive of hemangioma after biopsy. Bronchial hemangioma can cause clinical manifestations such as cough, hemoptysis and dyspnea in patients. Narita et al 2009, reported cases of bronchial hemangioma in all age groups in Japan with a mean age of 53 years. Men and women were affected to the same extent. 83% of patients had mild to severe hemoptysis as the main symptom.^[3]^

The treatment of bronchial hemangioma has not been established, and the main treatment methods are surgical resection (pulmonary wedge resection, lobectomy), bronchial artery ligation, and bronchial artery embolization (BAE). BAE has the advantages of minimal invasiveness, short operative time, and preservation of lung function. BAE was first described by the scholar Rémy in 1974 and has been used for benign and malignant causes of hemoptysis.^[4]^ According to a systematic review conducted by Panda, the clinical success rate of BAE is 70% to 99%.^[5]^

## 2. Clinical information

**Figure 1.**
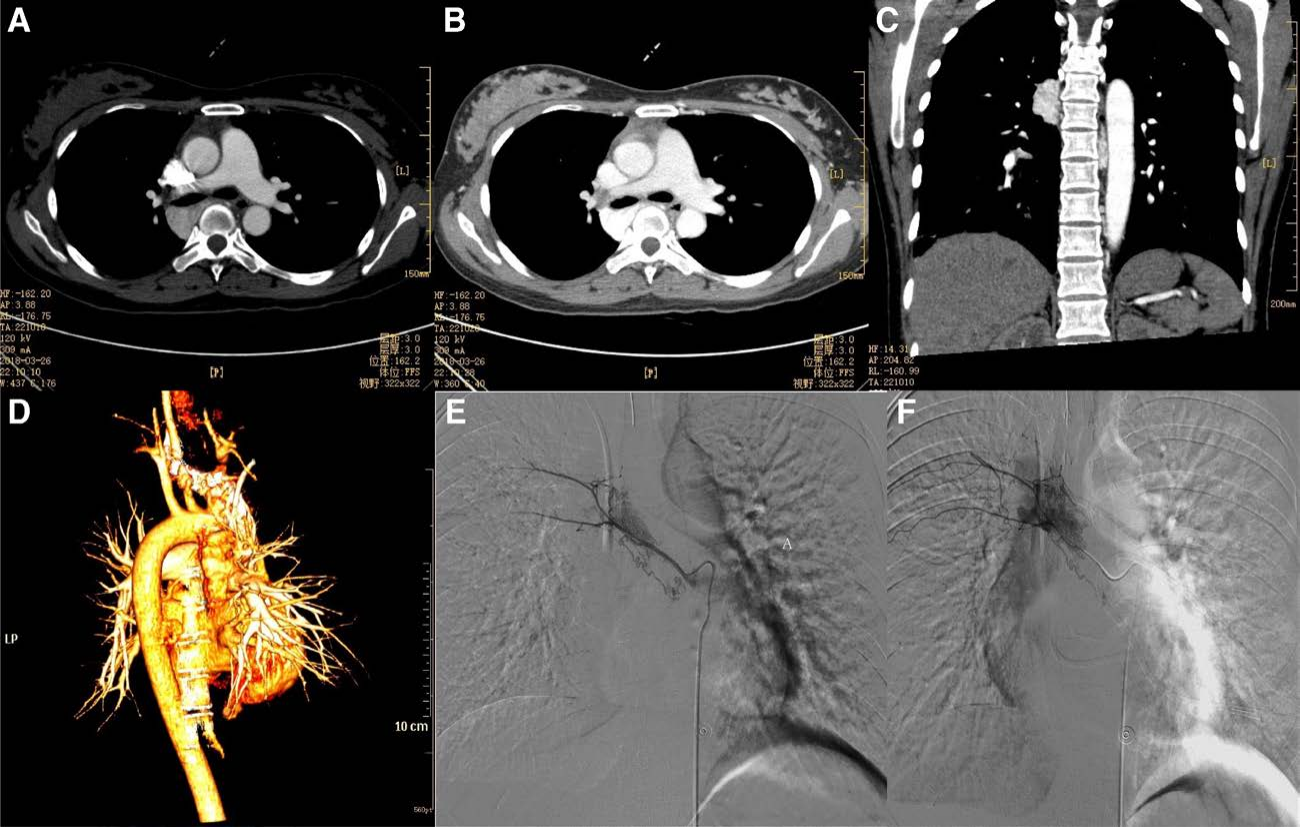
The imaging manifestation of the hemangioma and adjacent arteries for the first interventional therapy on March 27, 2018. (A and B) A mass of vascular-like enhancing shadow was seen behind the superior vena cava and around the right main bronchus, which was considered as a hemangioma (transverse section). (C) Sagittal section. (D) Three-dimensional reconstruction shows the morphology of the hemangioma. (E and F) Intraoperative DSA angiography shows that the right 6th and 7th intercostal arteries are involved in the blood supply of the hemangioma.

Patient, female, 26 years old, was admitted to the hospital on March 27, 2018 with “hemoptysis for 4 months, recurrence for 1 day.” Physical examination: body temperature: 36.5°C, pulse: 92 beats/min, respiration: 22 beats/min, blood pressure: 124/64 mm Hg, oxygen saturation:96%. Physical examination: clear consciousness, coarse respiratory sounds in both lungs, wet rales can be heard in the right lung, uniform heart rate, no murmur. The abdomen was flat and soft, with no pressure pain or rebound pain throughout the abdomen. Admission-related tests: hemoglobin 113g/L, platelet count 229 × 10^9^, prothrombin time 10.8 seconds. Enhanced CT examination of the lungs: a mass of vascular-like enhancing shadow was seen behind the superior vena cava in the mediastinum and around the right main bronchus, with a maximum cross-sectional size of 30 mm × 28 × 47 mm, which was mostly considered as a hemangioma (Fig. 1). After admission, the patient was treated with symptomatic treatment such as oxygen, hemostasis, control of gastric acid secretion and gastric protection. On the day of admission, the patient had a sudden hemoptysis of 300 ml, oxygen saturation dropped to 82%, pulse rate 124 beats/min, respiration 32 breaths/min, and blood pressure 104/75 mm Hg. She was transferred to the ICU for symptomatic supportive treatment with tracheal intubation and hemostasis. After full communication with the patient family, the patient was sent to the interventional procedure in an emergency. Intraoperatively, the right femoral artery sheath was locally anesthetized with 0.2% lidocaine, and after successful puncture of the right femoral artery using the Selding technique, a 5 F catheter sheath was placed, 5 F CORBA, 5 F SIMMONS, 5 F YASHIRO, and 3.0 F microcatheters were introduced, and superselective cannulation was performed to the right 6th and 7th intercostal arteries (Fig. 1), and the right upper lung avascular mass staining was seen on imaging. Polyvinyl alcohol (PVA) particles (100–300um, 300–500um) were embolized into the right 6th and 7th intercostal arteries, and the embolization was successful until the terminal artery was occluded. Re-selective cannulation to the adjacent intercostal artery, phrenic artery, right and left subclavian arteries, and abdominal trunk showed no abnormality. After the operation, the femoral artery sheath was removed to stop bleeding by compression for 15 minutes, and pressure bandaging was applied. On the second day after surgery, hemoglobin was 105 g/L, platelet count was 220 × 10^9^, and prothrombin time was 10.9 seconds. Pulse rate was 90 beats/min, respiration was 16 breaths/min, blood pressure was 115/60 mm Hg, and oxygen saturation was 100%. The patient did not have any further hemoptysis after surgery and was discharged.

**Figure 2.**
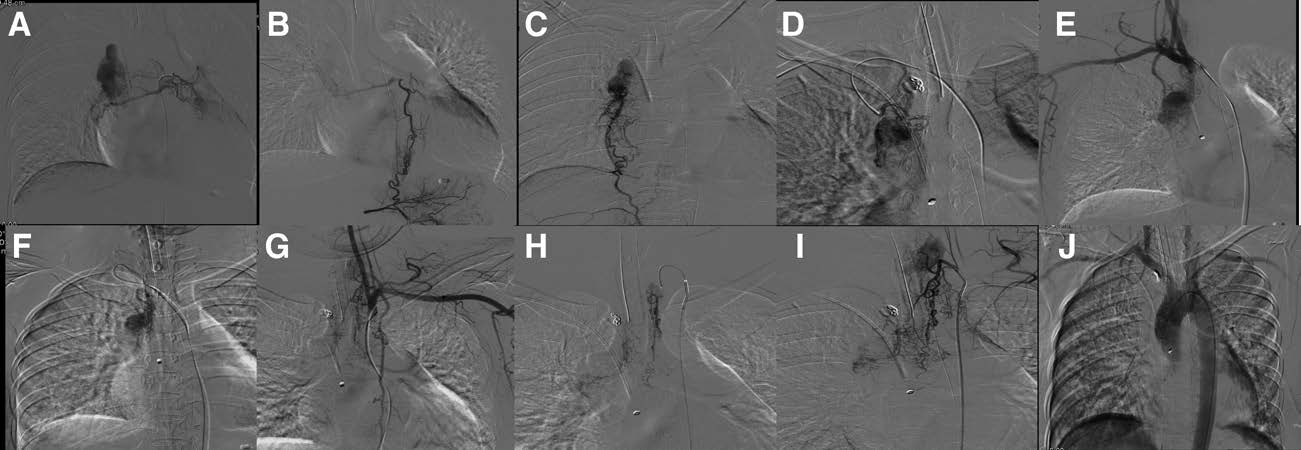
The bloody-supply situation with hemangioma for the second interventional therapy on July 27, 2018. (A) Bronchial artery involved in hemangioma blood supply. (B) Left gastric artery branch involved in hemangioma blood supply. (C) Right phrenic artery branch involved in hemangioma blood supply. (D) Right subclavian artery angiogram shows hemangioma pattern. (E) Right internal mammary artery branch involved in hemangioma blood supply. (F) Right thyrocervical trunk branch involved in hemangioma blood supply. (G) Left subclavian artery angiogram shows hemangioma pattern. (H) Left internal mammary artery branch involved in hemangioma blood supply. (I) Left thyrocervical trunk branch involved in hemangioma blood supply. (J) No hemangioma staining on postoperative aortogram.

The patient was admitted to the hospital on July 27, 2018 with “sudden hemoptysis for 1 hour.” Physical examination: pulse 135 beats/min, respiration 35 beats/min, blood pressure 154/93 mm Hg, oxygen saturation 85%. Physical examination: clear consciousness, coarse breath sounds in both lungs, wet rales could be heard in both lungs, and a uniform heart rate with no murmurs. The abdomen was flat and soft, and there was no pressure pain or rebound pain throughout the abdomen. Admission-related tests: hemoglobin 93g/L, platelet count 174 × 10^9^, prothrombin time 11.5 seconds. The patient was admitted with persistent hemoptysis of more than 500 mL, and was treated with tracheal intubation, hemostasis, and symptomatic support with fluid replacement. The consultation of respiratory medicine, cardiothoracic surgery and interventional medicine was comprehensive. Based on the size, nature and location of the lesion, it was judged that the surgical procedure was difficult and risky. The patient family discussed and requested interventional treatment. The patient was sent to the interventional unit for emergency treatment. After local anesthesia with 0.2% lidocaine, the right femoral artery was successfully punctured using the Selding technique, and a 5 F catheter sheath was placed, and catheters such as 5 F CORBA, 5 F single-curved catheter, 5 F YASHIRO, and 3.0 F microcatheter were introduced, and superselective cannulation to the right bronchial artery, bilateral subclavian arteries (bilateral metacervical trunk arteries, bilateral internal mammary arteries), and right phrenic artery (Fig. 2) was performed, and the above branches were seen to supply the right upper pulmonary vascular mass. The artery supplying the right upper pulmonary vascular mass was embolized with PVA particles (50um, 100–300um, 300–500um) and coils (2mm, 3mm, 4mm). No collateral vessels were seen on postoperative aortogram. At the end of the operation, the femoral sheath was removed to stop bleeding by compression for 15 minutes, and pressure bandaging was applied. On the second postoperative day, hemoglobin 82 g/L, platelet count 166 × 10^9^, and prothrombin time 10.6 seconds were rechecked. Pulse rate was 95 beats/min, respiration was 20 breaths/min, blood pressure was 120/70 mm Hg, and oxygen saturation was 98%. The patient had no further postoperative hemoptysis and was discharged successfully.

**Figure 3.**
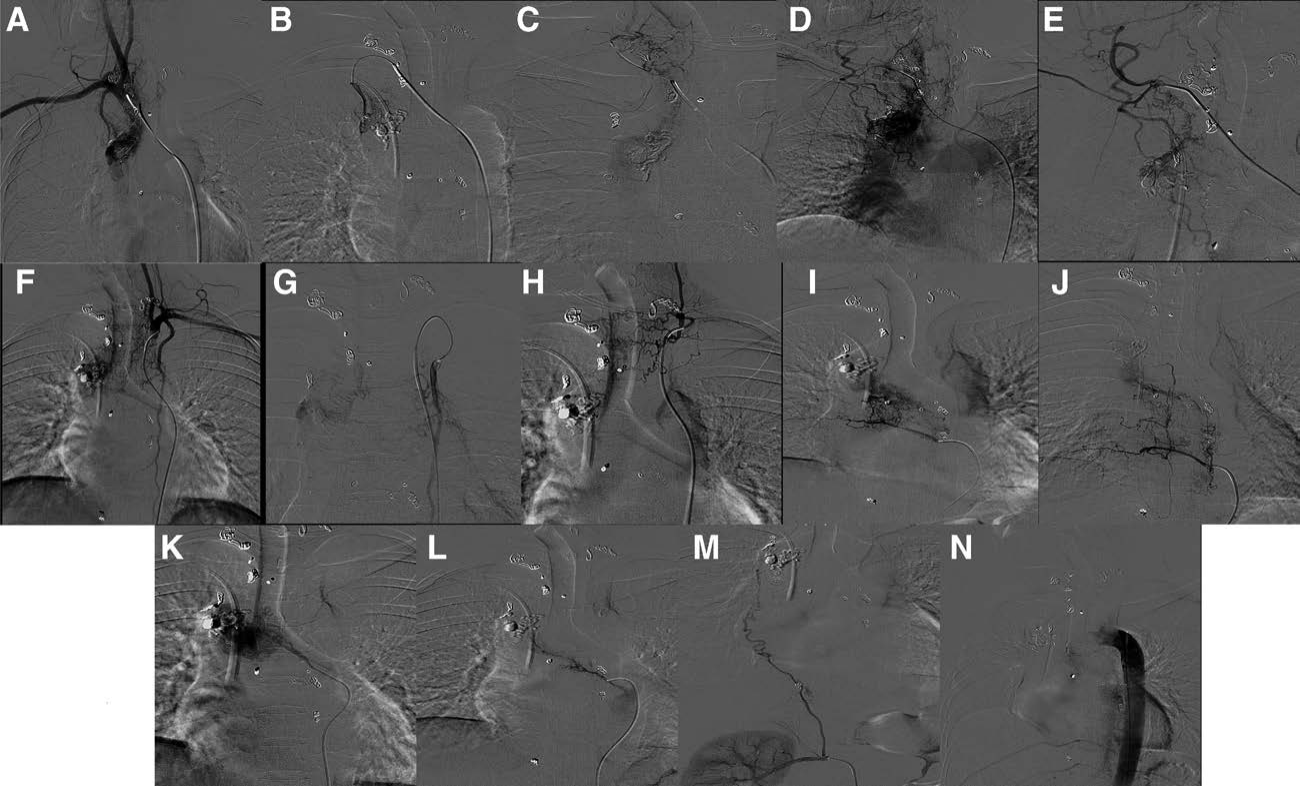
The intra-operative imaging manifestation-related to the bloody-supply situation with hemangioma for the third interventional therapy on October 01, 2019 (A). Right subclavian artery angiogram showing hemangioma pattern. (B) Right internal mammary artery branch involved in hemangioma blood supply. (C) Right thyrocervical trunk branch involved in hemangioma blood supply. (D). Hemangioma supply artery-pulmonary artery fistula formation. (E) Right supra-scapular artery branch involved in hemangioma blood supply. (F) Left subclavian artery angiogram showing hemangioma pattern. (G) Left internal mammary artery branch involved in hemangioma blood supply. (H) Left thyrocervical trunk branch involved in hemangioma blood supply. (I) The right 6th intercostal artery branch involved in hemangioma blood supply. (J) The right 7th intercostal artery branch involved in hemangioma blood supply. (K) Bronchial artery involved in hemangioma blood supply. (L) Bronchial artery involved in hemangioma blood supply. (M) Right renal artery branch involved in hemangioma blood supply. (N) No hemangioma staining on postoperative aortogram.

**Figure 4.**
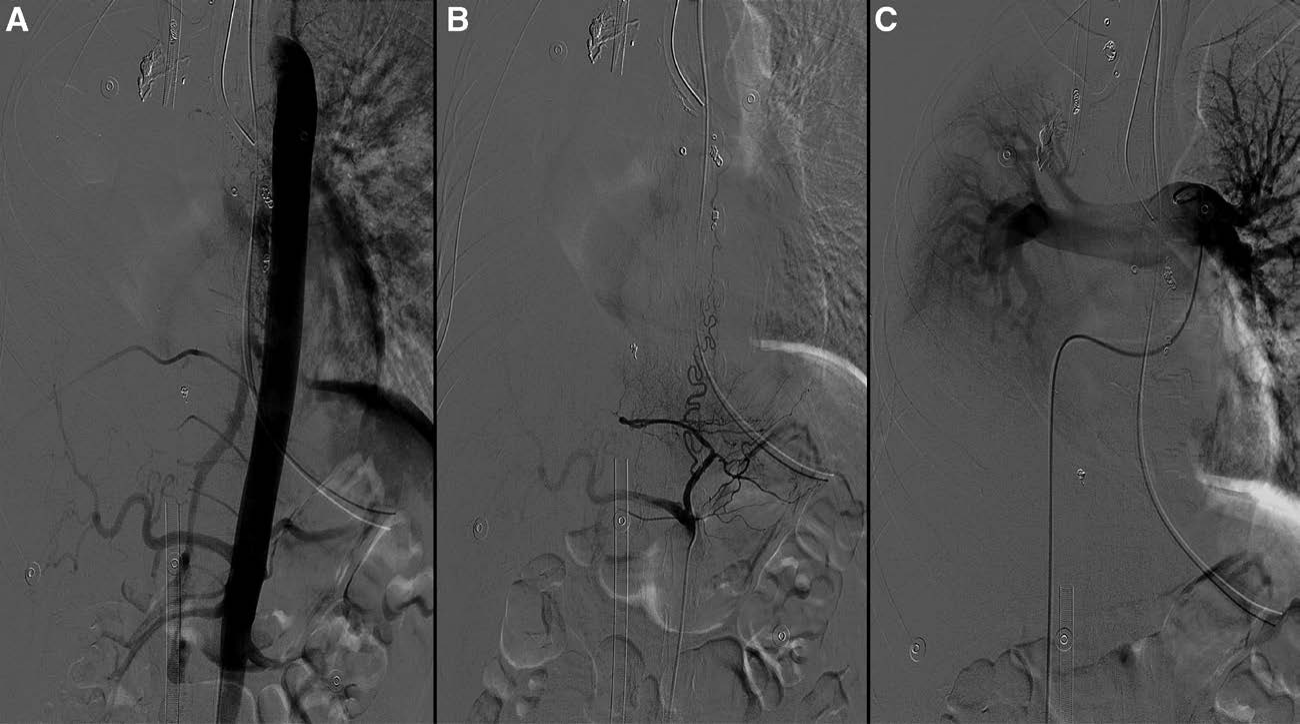
The radiography imaging of the hemangioma for the recurrent hemoptysis after 15-d off the third interventional therapy. (A). No hemangioma staining on aortography. (B) Left gastric artery branch involved in hemangioma blood supply. (C) Pulmonary arteriogram without hemangioma staining.

**Figure 5.**
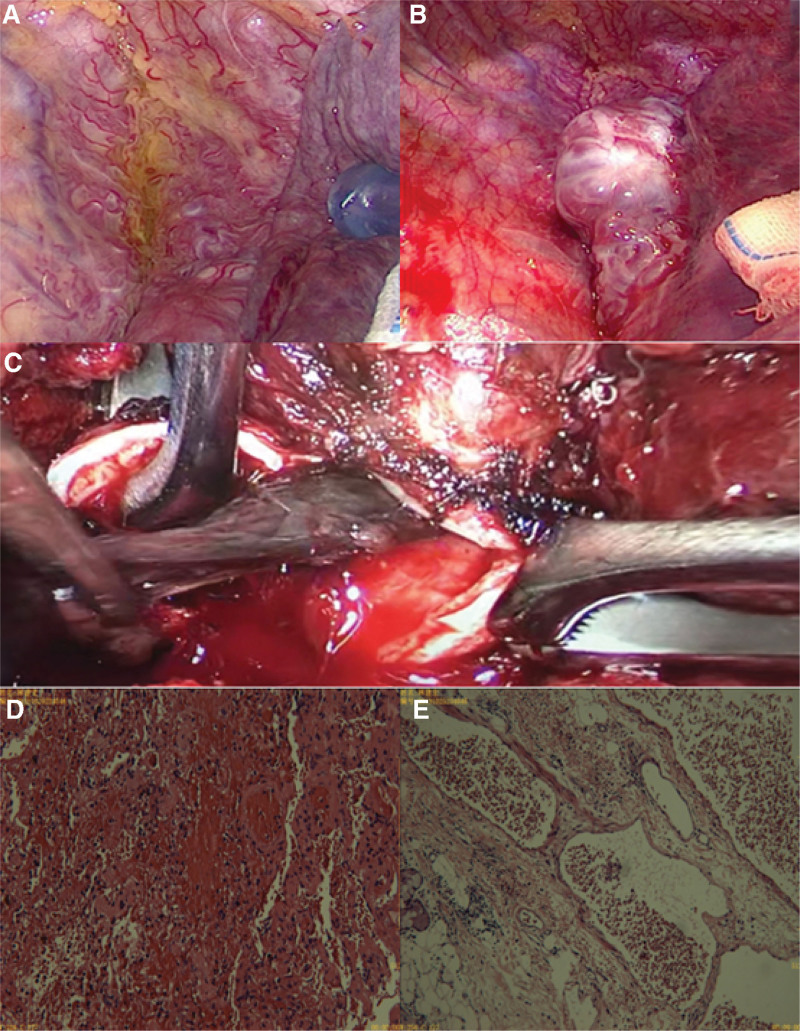
The biopsy results of the hemangioma with the surgical resection. (A) Massive abnormal vascular hyperplasia in the right upper mediastinum. The normal mediastinum pleura was not visualized. (B) The hemangioma was composed of a large number of criss-cross arteriovenous masses. (C) Intraoperatively, the right intermediate bronchus was opened. Several blood clots can be seen blocking the bronchus. The blood clots in the main airway were completely removed. (D and E) Microscopic examination shows vessels with uneven wall thickness, vascular dilatation and congestion with hemorrhage, lymphoid tissue hyperplasia, and no heterogeneous proliferation in the alveolar epithelium.

On October 01, 2019, the patient was admitted to the hospital with “hemoptysis for half a day.” The patient was admitted to the hospital with intermittent hemoptysis of about 10 ml each time. Physical examination: pulse 120 beats/min, respiration 20 beats/min, blood pressure 111/66 mm Hg, oxygen saturation 98%. Physical examination: clear consciousness, coarse respiratory sounds in both lungs, a small number of wet rales could be heard in the right lung, and a uniform heart rate with no murmur. The abdomen was flat and soft, and there was no pressure pain or rebound pain throughout the abdomen. Admission-related tests: hemoglobin 95g/L, platelet count 194 × 10^9^, prothrombin time 11.4 seconds. The patient family refused surgical treatment even after consultation with the Department of Respiratory Medicine, Cardiothoracic Surgery and Interventional Medicine. The patient was then sent to the interventional unit for emergency treatment. After successful puncture of the right femoral artery with the Selding technique, a 5 F catheter sheath was placed and 5 F CORBA, 5 F single-curved catheter, 5 F YASHIRO, 3.0 F microcatheter and other catheters were introduced and super-selectively cannulated to the right bronchial artery, bilateral subclavian arteries (bilateral metacervical trunk arteries, bilateral internal mammary arteries), right 6th and 7th intercostal arteries, right renal artery, right suprascapular artery, right phrenic artery (Fig. 3). The right upper pulmonary vascular mass was supplied with blood from the above branches, and a bronchial angioma donor artery-pulmonary artery shunt was seen. The right upper pulmonary vascular mass donor artery was embolized with PVA particles (50–100um, 100–300um, 300–500um), coils (2 mm, 3 mm, 4 mm) and N-butyl-2-cyanoacrylate (NBCA). No collateral vessels were seen on postoperative aortogram. At the end of the operation, the femoral sheath was removed to stop bleeding by compression for 15 minutes, and pressure bandaging was applied. On the 15th postoperative day, the patient had a recurrent hemoptysis of 300 mL, a pulse rate of 130 beats/min, 33 breaths/min, a blood pressure of 157/96 mm Hg, and an oxygen saturation of 79%. She was urgently intubated and transferred to the ICU for symptomatic support treatment. Aortogram and pulmonary arteriogram were performed, and only the branch vessels of the left gastric artery supplying the hemangioma were visible. PVA pellets (100–300 µm) were given for embolization (Fig. 4). Postoperatively, the patient continued to hemoptysis. The patient arterial oxygen saturation decreased to 70%, heart rate increased to > 150 beats/min, and blood pressure decreased to 70/40 mm Hg. Extracorporeal Membrane Oxygenation was then initiated. Emergency surgery followed. Intraoperatively, a bronchial hemangioma with diffuse abnormal vascular growth in the right mediastinal pleura was seen. The base of the hemangioma was located in the right main bronchus, and the abnormal vascular plexus extended into the right upper lobe bronchus and middle bronchus. Right upper lobe and bronchial hemangioma resection was performed (Fig. 5). There was no further bleeding from the airway after the operation, and extracorporeal membrane oxygenation was removed 48 hours after the operation, and the patient was subsequently extubated and transferred to a general ward. The patient has been followed up for 4 years without recurrence of hemoptysis.

## 3. Discussion

The embolism materials for BAE include gelatin sponge, PVA, coils, and N-butyl-2-cyanoacrylate (NBCA).^[6]^ Among them, gelatin sponge is a temporary embolic agent, which is absorbed within 2 to 6 weeks and can make possible the reopening of embolized vessels, and is now less commonly used. PVA, coils and NBCA are permanent embolic agents and are more widely used in BAE. However, if PVA or coils are not adequately placed at the bleeding source of the hemangioma, they may open the hemangioma collateral blood supply arteries and cause rebleeding in the short term. NBCA is a liquid embolic material that coagulates rapidly in contact with blood after injection into the body and has a better effect on the embolization of the vascular nest of the hemangioma. Then, improper injection practice can lead to influx of NBCA into the lung or systemic circulation, causing serious complications. Therefore, a combination of coil/PVA and NBCA is recommended to obtain a more solid embolization effect. Surgical resection and bronchial artery ligation are also treatments for bronchial hemangiomas, but these methods are associated with higher complications and mortality. Surgical resection allows complete removal of the hemangioma without risk of rebleeding even in complicated cases where bronchial artery-pulmonary artery fistula and collateral circulation are present, but it is a very invasive approach and decreases lung function. Bronchial artery ligation is sometimes an option to avoid loss of lung function. However, it does not prevent the establishment of collateral circulation by the angioma causing rebleeding. In this case, the patient still had bleeding after multiple BAE, and the author considered the following reasons: Incomplete embolization of the vascular nest of the hemangioma, which caused vascular proliferation and opening of the collateral circulation, causing the hemangioma to continue to grow; Anastomosis or fistulization of the hemangioma with the pulmonary artery or vein, and the presence of bronchial artery-pulmonary artery fistula is a risk factor for massive hemoptysis, because the bronchial artery is supplied by the systemic circulation, and its pressure and velocity are higher than those of the pulmonary artery, making the pressure and blood flow rate in the vascular nest of the angioma high.^[7,8]^

Bronchial hemangiomas in adults are extremely rare, and laboratory tests often do not reveal abnormal findings. Patients with hemoptysis as a clinical manifestation should be considered as a possible disease, and the diagnosis should be clarified with enhanced CT or fibronectomy, and biopsy should be performed with caution. For bronchial angiomas without bronchial artery-pulmonary arteriovenous fistula, we recommend BAE as the first choice of treatment. For bronchial angiomas with bronchial artery-pulmonary arteriovenous fistulae, we recommend early surgical resection if BAE is ineffective. Clinicians should improve their knowledge of bronchial hemangiomas to avoid missed and misdiagnosis.

## Acknowledgments

Sincerely thanks for the root of patient and her relatives for the whole treatment and the support of Prof Jian Zhang.

## Author contributions

**Conceptualization:** Zhipeng Lin.

**Formal analysis:** Xugong Zou.

**Investigation:** Zhipeng Lin.

**Methodology:** Zhipeng Lin, Xiaolong Hu, Yuan Chen.

**Project administration:** Jian Zhang.

**Resources:** Jian Zhang.

**Validation:** Xugong Zou, Yuan Chen, Xiaoqun Li.

**Visualization:** Xiaolong Hu.

**Writing – original draft:** Zhipeng Lin.

**Writing – review & editing:** Xiaoqun Li, Dabei Huang.
